# Author Correction: A shuttling-based two-qubit logic gate for linking distant silicon quantum processors

**DOI:** 10.1038/s41467-022-34236-2

**Published:** 2022-11-01

**Authors:** Akito Noiri, Kenta Takeda, Takashi Nakajima, Takashi Kobayashi, Amir Sammak, Giordano Scappucci, Seigo Tarucha

**Affiliations:** 1 RIKEN Center for Emergent Matter Science (CEMS), Wako, Japan; 2 RIKEN Center for Quantum Computing (RQC), Wako, Japan; 3 QuTech, Delft University of Technology, Delft, The Netherlands; 4 Netherlands Organization for Applied Scientific Research (TNO), Delft, The Netherlands; 5 Kavli Institute of Nanoscience, Delft University of Technology, Delft, The Netherlands

**Keywords:** Qubits, Quantum information, Quantum dots

Correction to: *Nature Communications* 10.1038/s41467-022-33453-z, published online 30 September 2022

The original version of this Article contained an error in a sentence in the last paragraph of the Introduction, which incorrectly read ‘The shuttling-mode exchange switching allows us to efficiently control exchange coupling with an on/off ratio above 1000,000.’ The correct version states ‘1000’ in place of ‘1000,000’.

This has been corrected in the PDF version of the Article.

