# Supplementary material for: A shuttling-based two-qubit logic gate for linking distant silicon quantum processors
Source: Nat Commun. 2022 Sep 30;13:5740. doi: 10.1038/s41467-022-33453-z (PMC9525571; doi:10.1038/s41467-022-33453-z)
Supplement: Supplementary file 1 — Supplementary Information [file 41467_2022_33453_MOESM1_ESM.pdf]

Supplementary Information for

A shuttling-based two-qubit logic gate for linking distant silicon quantum processors

Akito Noiri[1,*], Kenta Takeda[1], Takashi Nakajima[1], Takashi Kobayashi[2], Amir Sammak[3,4], Giordano Scappucci[3,5], and Seigo Tarucha[1,2,*]

[1]*RIKEN Center for Emergent Matter Science (CEMS), Wako, Japan*

[2]*RIKEN Center for Quantum Computing (RQC), Wako, Japan*

[3]*QuTech, Delft University of Technology, Delft, The Netherlands*

[4]*Netherlands Organization for Applied Scientific Research (TNO), Delft, The Netherlands*

[5]*Kavli Institute of Nanoscience, Delft University of Technology, Delft, The Netherlands*

[*]e-mail: akito.noiri@riken.jp or tarucha@riken.jp

Supplementary Note 1: Sample fabrication.

The triple quantum dot is defined at the isotopically enriched silicon quantum well (residual ^{29}silicon concentration of 800 parts per million). The device is identical to the one used in Ref. 1. The separation between the center of quantum dots and the plunger gate width is ~ 90 nm and ~ 65 nm, respectively. The small (~ 25 nm) gap between the plunger gates makes the control of inter-dot tunnel coupling by the barrier gate inefficient and a large (> 1 V) positive voltage is required to achieve sufficiently large inter-dot tunnel couplings t_{R} and t_{L} simultaneously for high-fidelity electron shuttling and for inducing a large J. We also find that the device becomes unstable if the barrier gate voltage exceeds 1 V, which limits the available range of J in this device. We note that barrier gate pulses reduce the requirement of making t_{R} and t_{L} large simultaneously, but we cannot use them in this work due to the limitation of the number of outputs of the arbitrary waveform generator used (see Methods). We anticipate that, by increasing the width of the barrier gates, t_{L} and therefore J can be efficiently modified by the barrier gate pulse with which a larger J will be available around the charge-symmetry point.

Supplementary Note 2: Simulation of EDSR frequency detuning of single-qubit gate fidelity

We simulate the effect of residual exchange coupling on the single-qubit primitive gate fidelity as it is one of the most relevant sources of a gate crosstalk[2–4] which needs to be avoided for scaling up. Under a finite exchange coupling, resonance frequency of a qubit depends on the state of the other qubit, making a drive of single-qubit gate slightly off-resonant. Therefore, we discuss how the single-qubit gate fidelity depends on EDSR frequency detuning in this section. The Hamiltonian of the single-qubit system in a rotating frame with a frequency of applied microwave can be described by $H_{\mathrm{R}} = \frac{h}{2}\begin{pmatrix} \delta f & f_{\mathrm{R}}\mathrm{e}^{i\phi} \\ f_{\mathrm{R}}\mathrm{e}^{-i\phi} & -\delta f \end{pmatrix}$ where h is the Planck's constant, δf is the frequency difference between the microwave and the EDSR resonance condition, f_{R} is the Rabi frequency, and ϕ is the phase of the microwave. We calculate $\pi/2$ rotation operators by $\mathrm{U} = \prod_{\mathrm{k=0}}^{\mathrm{k=N}} \mathrm{e}^{-\mathrm{i}2\pi H_{\mathrm{R}}\mathrm{k}\Delta t/h}$ where $\Delta t = t_{\mathrm{hp}}/\mathrm{N}$, $t_{\mathrm{hp}} = 1/(4f_{\mathrm{R}})$, and N is a large integer ($\mathrm{N} = 1{,}000$ in our calculation) and obtain operators of all the Clifford gates. Then we calculate the probability of the ideal final state as a function of the number of randomly chosen Clifford gates with $L = 1, 11, 21, \ldots, 101$. We average 1,000 random sequences to obtain the sequence fidelity. Then we extract the single-qubit primitive gate fidelity from the sequence fidelity by the above procedure. Supplementary Figure 9 shows δf dependence of the single-qubit primitive gate fidelity. $\delta f = 100$ kHz is too large to keep the demonstrated high-fidelity ($> 99.9\%$) of the single-qubit gate[5] with Rabi freuquency of a few MHz, which is typical to EDSR control of silicon spin qubits[1,2,4–6]. To maintain the demonstrated high-fidelity (99.98%[7]) of the single-qubit gate, the residual coupling needs to be decreased down to ~ 10 kHz. We actually obtain a sufficiently small residual J of 0.89 ± 0.1 kHz in the sparse state (Supplementary Fig. 6b). With

this condition, we also obtain a high-fidelity ($F_{\mathrm{p,s}} = 99.906 \pm 0.002\%$ for Q_L and $99.751 \pm 0.003\%$ for Q_M) single-qubit gates (Fig. 1f, g) with a Rabi frequency of 2.5 MHz even when the same gate sequence is applied to both qubits simultaneously.

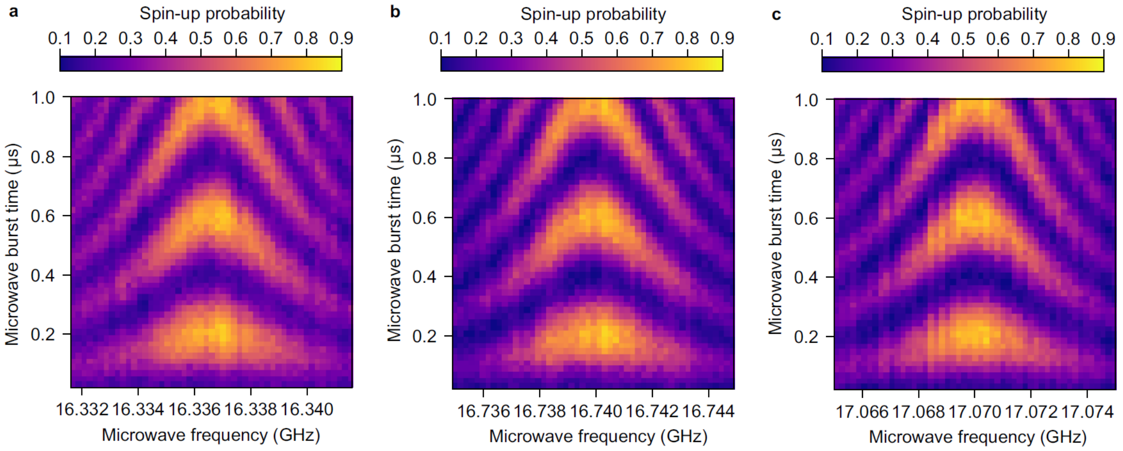

Supplementary Fig. 1. Rabi oscillations for a spin at each dot. a-c, Rabi oscillations of a spin in the left dot in **a**, center dot in **b**, and right dot in **c**. To measure the Rabi oscillation in **a** (**c**), Q_L (Q_M) is manipulated in the sparse state at the white square labeled C in Fig. 1c. To measure **b**, Q_M is initialized at the right dot, moved to the center dot and manipulated in the coupled state at the white star labeled D in Fig. 2b and Fig. 3a. Here Q_L is always spin-down. Then it is moved back to the right dot and measured. The resonance frequency is 16.3366 GHz (**a**), 16.7399 GHz (**b**), and 17.0700 GHz (**c**), respectively. Rabi frequencies are tuned to be 2.5 MHz.

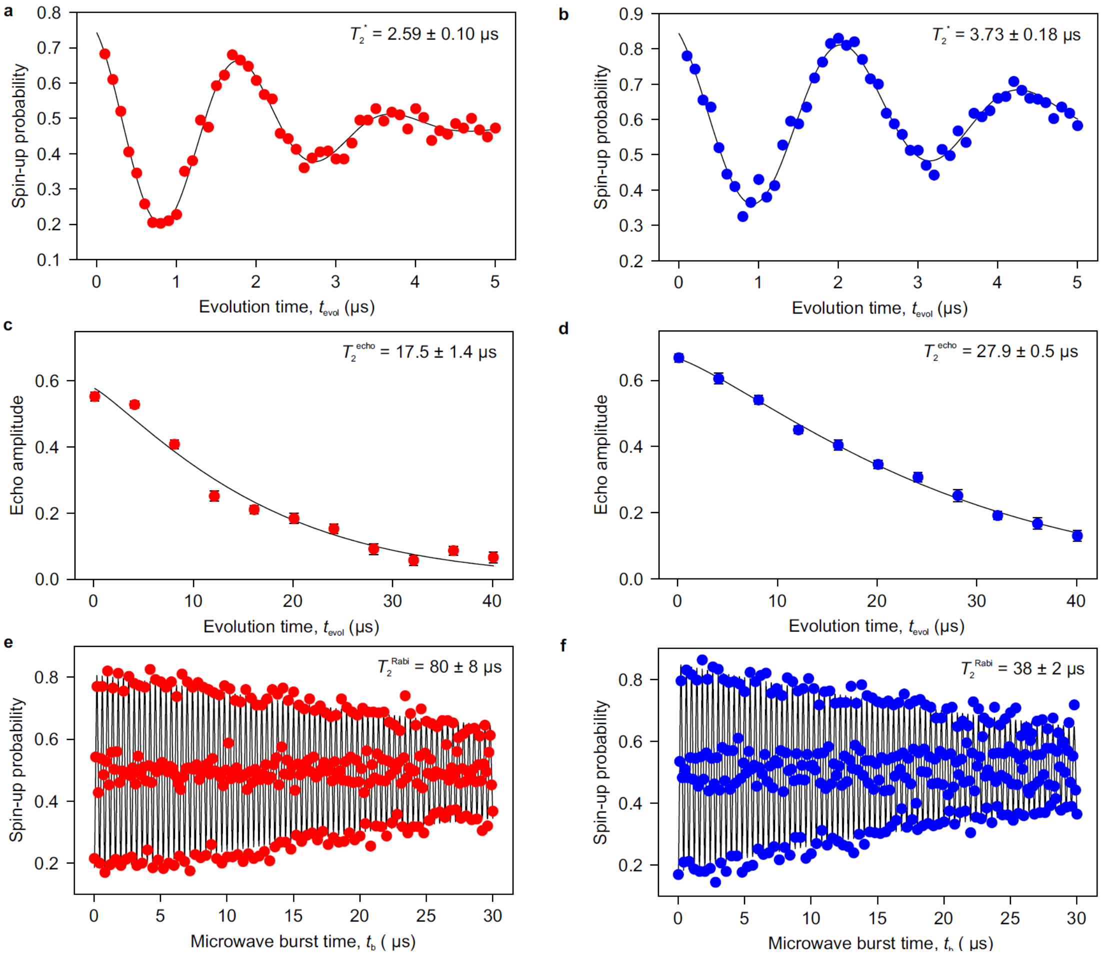

Supplementary Fig. 2. Single-qubit performance in the sparse state. a, b, Ramsey fringe with a fit of Gaussian decaying oscillation function for Q_L (**a**) and Q_M (**b**), respectively. The data acquisition time is 1.2 minutes for each trace. Although the device is identical to the one used in ref. 1, we obtain a shorter dephasing time T_2^* for both qubits than those measured in ref. 1 possibly due to increased charge noise in the gate voltage condition used in this work (Supplementary Fig. 3). All the measurements are performed in the sparse state. Also, the same sequence is applied to both qubits simultaneously and Q_M is measured followed by Q_L readout. The errors represent the estimated standard errors for the best-fit values. **c, d,** Decay of the echo amplitude[1,4] as a function of the evolution time for characterizing an echo time T_2^{echo}. The echo amplitude is obtained by varying the phase of the final $\pi/2$ rotation. The exponent of the decay is 1.1 for **c** (Q_L) and 1.3 for **d** (Q_M). The errors represent the estimated standard errors for the best-fit values. **e, f,** Rabi oscillation for Q_L in **e** and Q_M in **f**. The oscillation decay follows $\exp\left(-t_b/T_2^{\text{Rabi}}\right)W(t_b, f_R)$ where t_b is the MW burst time, T_2^{Rabi} is the decay time of Rabi oscillation, f_R is the Rabi frequency, and $W(t_b, f_R) = (1 + t_b{}^2/(f_R(T_2^*)^2)^2)^{-1/4}$ represents the effect of dephasing[8]. The errors represent the estimated standard errors for the best-fit values.

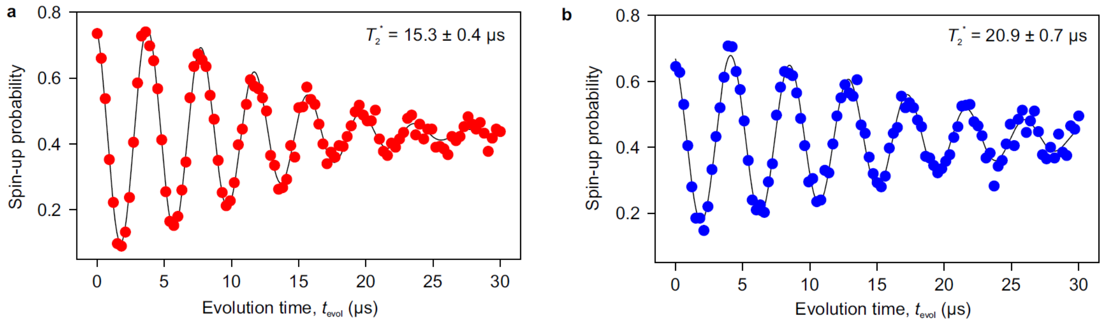

Supplementary Fig. 3. Single-qubit dephasing times in the sparse state under weak inter-dot tunnel couplings. a, b, Ramsey fringe with a fit of Gaussian decaying oscillation function for Q_L (**a**) and Q_M (**b**), respectively. The data acquisition time is 2.1 minutes for each trace. The errors represent the estimated standard errors for the best-fit values. The measurements are performed in the sparse state. Compared to the measurements in Supplementary Fig. 2a, b, the barrier gate voltages which control t_R and t_L are decreased by 400 mV. We find that T_2^* for both qubits depend on the barrier gate voltages and larger barrier gate voltages result in shorter T_2^* as shown in Supplementary Fig. 2a, b. This is most likely because charge noise, whose magnitude depends on the gate voltage condition, couples to the qubits under the field gradient created by the micromagnet, enhancing dephasing of the qubits[5].

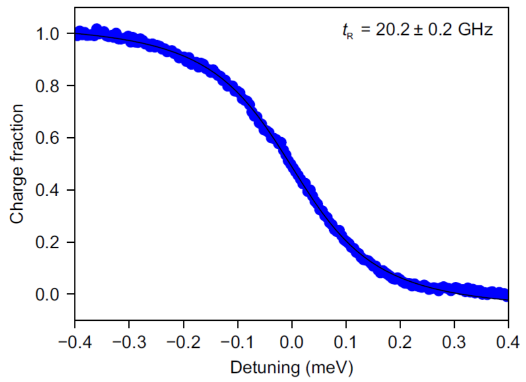

Supplementary Fig. 4. Evaluation of inter-dot tunnel coupling between center and right dot. Charge transition between (1,1,0) and (1,0,1) charge states with the fitting curve[9]. The errors represent the estimated standard errors for the best-fit values.

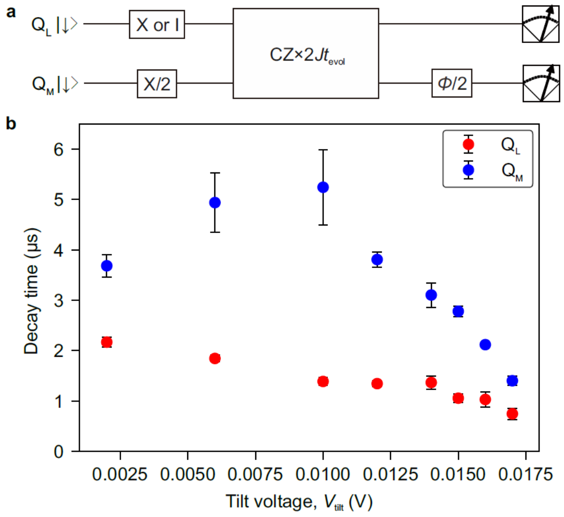

Supplementary Fig. 5. Tilt voltage dependence of T_2^*. a, Pulse sequence to produce **b**. The sequence is similar to the one shown in Fig. 3c but the π rotation for both qubits in the middle of the exchange gate is omitted. **b,** Operation point dependence of T_2^* which is smaller than that obtained with the decoupled sequence (Fig. 3d). The errors represent the estimated standard errors for the best-fit values.

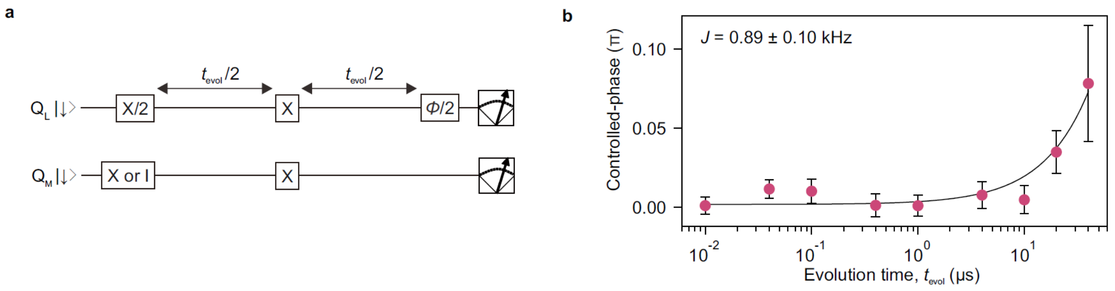

Supplementary Fig. 6. Residual exchange coupling in the sparse state. a, Quantum circuit to produce **b**. The π rotations for both qubits in the middle of the phase evolution decouples quasi-static noise[3]. Similar to Fig. 3c, we measure the controlled phase of $2\pi J t_{\mathrm{evol}}$ accumulated in Q_L during the evolution time of t_{evol} and obtain J. **b,** Residual J measured in the sparse state. The errors represent the estimated standard errors for the best-fit values.

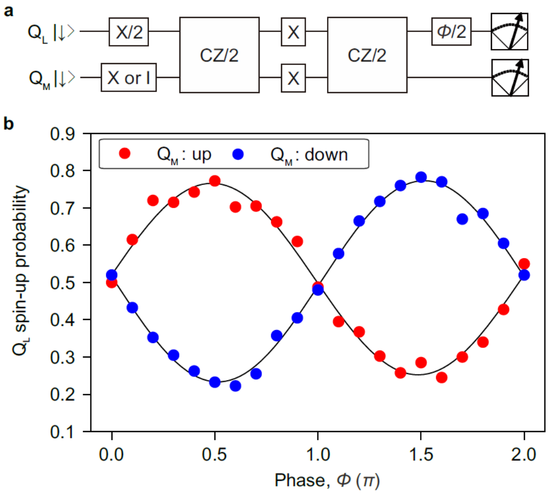

Supplementary Fig. 7. Calibration of unconditional phase accumulation in Q_1 in DCZ gate. a, Quantum circuit used for calibrating the unconditional phase accumulation in Q_L during the DCZ gate. **b,** Accumulated phase in Q_L in the DCZ gate when Q_M is prepared in spin-down (blue) and -up (red) measured using the circuit shown in **a**. Here, the unconditional phase accumulation of 0.065π for Q_L is compensated by shifting the phase of the final $\pi/2$ rotation[3,4,10].

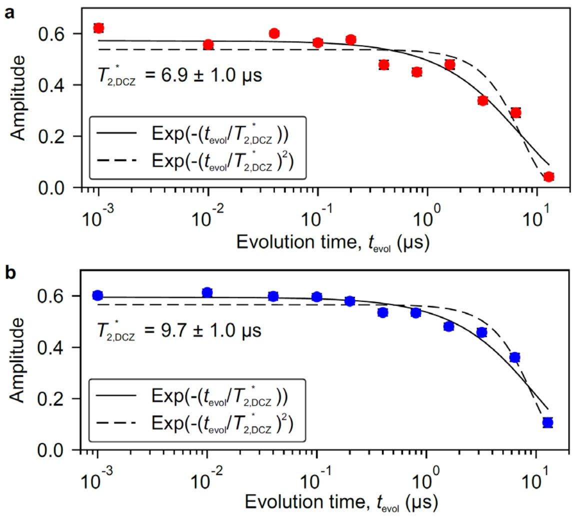

Supplementary Fig. 8. Decoupled qubits dephasing. a, b, Decay property for Q_L (**a**) and Q_M (**b**) measured with the quantum circuit shown in Fig. 3c plotted with exponential decay (the solid line) and Gaussian decay (the broken line). $V_{\text{tilt}} = 0.012$ V is used. The errors represent the estimated standard errors for the best-fit values. For both qubits, the exponential decay seems to fit the data better than the Gaussian decay[3]. If we fit the data to $a\exp\left(-\left(t_{\text{evol}}/T^*_{2,\text{DCZ}}\right)^n\right)$ where a is the amplitude, we obtain $n = 0.9 \pm 0.2$ for Q_L and 1.2 ± 0.2 for Q_M, respectively. Therefore, we obtain the decoupled dephasing time $T^*_{2,\text{DCZ}}$ by fitting the data to the exponential decay. This suggests that the effect of dephasing during the controlled-phase accumulation on the CZ gate is roughly $\mathrm{e}^{-(0.4/7)} =$ 94.5%. This indicates the most part of the error in our CZ gate comes from dephasing during the controlled-phase accumulation.

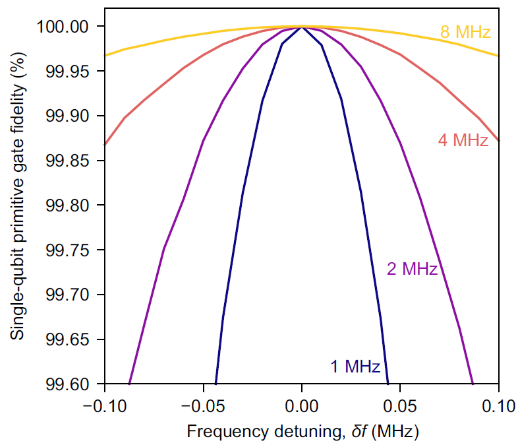

Supplementary Fig. 9. Simulated frequency detuning dependence of single-qubit gate fidelity. Simulated single-qubit primitive gate fidelity as a function of frequency detuning (see Supplementary Note 2 for the calculation procedure). Rabi frequency is 1 MHz, 2 MHz, 4 MHz, and 8 MHz for the indigo, purple, orange, and yellow trace, respectively.

Supplementary References:

1. Noiri, A. *et al.* Fast universal quantum gate above the fault-tolerance threshold in silicon. *Nature* **601**, 338–342 (2022).
2. Zajac, D. M. *et al.* Resonantly driven CNOT gate for electron spins. *Science* **359**, 439–442 (2018).
3. Watson, T. F. *et al.* A programmable two-qubit quantum processor in silicon. *Nature* **555**, 633–637 (2018).
4. Takeda, K. *et al.* Quantum tomography of an entangled three-qubit state in silicon. *Nat. Nanotechnol.* **16**, 965–969 (2021).
5. Yoneda, J. *et al.* A quantum-dot spin qubit with coherence limited by charge noise and fidelity higher than 99.9%. *Nat. Nanotechnol.* **13**, 102–106 (2018).
6. Xue, X. *et al.* Quantum logic with spin qubits crossing the surface code threshold. *Nature* **601**, 343–347 (2022).
7. Yang, C. H. *et al.* Silicon qubit fidelities approaching incoherent noise limits via pulse engineering. *Nat. Electron.* **2**, 151–158 (2019).
8. Nakajima, T. *et al.* Coherence of a Driven Electron Spin Qubit Actively Decoupled from Quasistatic Noise. *Phys. Rev. X* **10**, 11060 (2020).
9. DiCarlo, L. *et al.* Differential charge sensing and charge delocalization in a tunable double quantum dot. *Phys. Rev. Lett.* **92**, 226801 (2004).
10. Huang, W. *et al.* Fidelity benchmarks for two-qubit gates in silicon. *Nature* **569**, 532–536 (2019).
